# Editorial excellence and impact on public health: a strategic look from scientific journals of the global south

**DOI:** 10.17843/rpmesp.2026.431.16258

**Published:** 2026-02-26

**Authors:** César Cabezas

**Affiliations:** 1 Instituto Nacional de Salud, Lima, Perú.

Scientific journals are undergoing a structural crisis that extends far beyond the technical aspects of academic publishing. This crisis is multidimensional and encompasses problems of financial sustainability, editorial governance, scientific integrity, global inequity in access to knowledge, and a progressive reduction of the value of science to bibliometric indicators, a current example of which is the quartile system ^1^. In this context, the pressure to publish in high-impact journals has favored questionable practices, such as the fragmentation of results, authorship inflation, the proliferation of so-called *paper mills*, and the increase in documented retractions globally ^2^; therefore, we must be aware of the problem and its solution.

In the field of public health, this threat is particularly dangerous, given that the impact of research is not limited to academic citation, but is expressed in public policies, health programs, management decisions, and concrete improvements in the health of the population.

In recent decades, the scientific editorial system has experienced an unprecedented expansion in the volume of manuscripts submitted for evaluation. In part, this growth has been driven by academic incentive systems focused on quantitative productivity, which privilege the number of publications over their social or health relevance. As a consequence, one of the most visible problems of the contemporary system is the so-called peer reviewer crisis; since there is an extremely high number of manuscripts to review, and as this is an unpaid activity, experts in different fields decline invitations to review manuscripts, which significantly lengthens editorial processes ^3^^).^

The reviewer crisis not only affects the efficiency of the editorial process but also poses risks to scientific quality. Peer review constitutes one of the fundamental pillars of the credibility of science, and its weakening can facilitate the publication of studies with methodological limitations, inadequate interpretations, or unsupported conclusions.

Added to these problems is the transformation of the open science model. Although open access to scientific knowledge constitutes a principle widely supported by the academic community and international organizations, its implementation has been dominated by *Article Processing Charges (APC)*. This model has shifted the cost of publication to authors or their institutions, deepening inequalities between countries and institutions with different resource levels ^4^^,^^5^.

The concentration of the publishing market in a small number of multinational corporations has reinforced these pressures. In this scenario, access, visibility, and scientific prestige are mediated by commercial logics that do not always align with public health priorities or the needs of low- and middle-income countries.

An emerging challenge that further tests the editorial system is the increasing use of artificial intelligence (AI) tools in scientific production. Generative AI can support writing or translation tasks, but its unregulated use has facilitated the generation of manuscripts with conceptual errors, non-existent references, and opacity in authorship. Furthermore, the use of AI in peer review raises questions about algorithmic biases and editorial responsibility ^6^.

From a public health perspective, the risk does not lie in the technology itself, but in its uncritical use within a system already strained by inadequate incentives. Faced with this, it is essential to move towards a reasonable and transparent use of AI ^6^.

On the other hand, the Peruvian context offers multiple examples of the lack of equivalence between real health impact and bibliometric visibility. Studies on hepatitis B and D conducted in the Amazon and high Andean areas contributed decisively to universal vaccination policies and the control of horizontal and vertical transmission, aligned with WHO recommendations ^7^^,^^8^.

During the COVID-19 pandemic, the development and validation of diagnostic tests allowed for the expansion of diagnostic capacity and the strengthening of epidemiological surveillance in real-time ^9^. Advances in the diagnosis of arboviruses such as dengue, anemia, and exposure to heavy metals have supported public health actions oriented toward territorial and environmental equity ^10^^,^^11^^,^^12^.

Similarly, research on antimalarial resistance guided therapeutic changes and strengthened the surveillance of pharmacological efficacy against malaria in the Peruvian Amazon ^13^. Studies on tuberculosis contributed to regulatory adjustments and the national programmatic response ^14^, as was the case with HIV/AIDS ^15^.

Studies on tuberculosis contributed to regulatory adjustments and the national programmatic response,

These examples show that the impact of public health research takes on regulatory, programmatic, and diagnostic forms that are not always captured by traditional bibliometric metrics.

Faced with this scenario, a dual evaluation of scientific journals is proposed, complementing bibliometric indicators with public health impact metrics, in line with international initiatives for the reform of research assessment ^16^.

International visibility remains a legitimate and necessary means to amplify the circulation of scientific knowledge. However, the ultimate goal of public health science must be to improve the health of populations and reduce inequities, integrating editorial excellence, scientific integrity, and the responsible use of new technologies.
