# Supplementary material for: Editorial excellence and impact on public health: a strategic look from scientific journals of the global south
Source: Rev Peru Med Exp Salud Publica. 2026 Feb 26;43(1):4–5. doi: 10.17843/rpmesp.2026.431.16258 (PMC13245982; doi:10.17843/rpmesp.2026.431.16258)
Supplement: Translation

# Translation: Excelencia editorial e impacto en salud pública: una mirada estratégica desde las revistas científicas del sur global

**Authors:** César Cabezas

**Affiliations:** 1 Instituto Nacional de Salud. Lima, Peru.

Las revistas científicas atraviesan una crisis estructural que trasciende ampliamente los aspectos técnicos de la publicación académica. Esta crisis es multidimensional y comprende problemas de sostenibilidad financiera, gobernanza editorial, integridad científica, inequidad global en el acceso al conocimiento y una progresiva reducción del valor de la ciencia a indicadores bibliométricos, cuyo ejemplo vigente es el sistema de quartiles ^1^. En este contexto, la presión por publicar en revistas de alto impacto ha favorecido prácticas cuestionables, como la fragmentación de resultados, la inflación de autorías, la proliferación de los denominados *paper mills* y el incremento de retractaciones documentadas a nivel global ^2^ por lo que debemos ser conscientes del problema y su solución.

En el campo de la salud pública, esta amenaza resulta particularmente peligrosa, dado que el impacto de la investigación no se limita a la citación académica, sino que se expresa en políticas públicas, programas sanitarios, decisiones de gestión y mejoras concretas en la salud de la población.

En las últimas décadas, el sistema editorial científico ha experimentado una expansión sin precedentes en el volumen de manuscritos sometidos a evaluación. En parte este crecimiento ha sido impulsado por sistemas de incentivos académicos centrados en la productividad cuantitativa, que privilegian el número de publicaciones por encima de su relevancia social o sanitaria. Como consecuencia, uno de los problemas más visibles del sistema contemporáneo es la denominada crisis de revisores pares, ya que al haber un número altísismo de manuscritos a revisar, y al ser esta una actividad no remunerada, los expertos en los diferentes campos declinan las invitaciones para revisar los manuscritos, lo cual alarga de manera significativa los procesos editoriales ^3^.

La crisis de revisores no solo afecta la eficiencia del proceso editorial, sino que plantea riesgos para la calidad científica. La evaluación por pares constituye uno de los pilares fundamentales de la credibilidad de la ciencia, y su debilitamiento puede facilitar la publicación de estudios con limitaciones metodológicas, interpretaciones inadecuadas o conclusiones no sustentadas.

A estos problemas se suma la transformación del modelo de ciencia abierta. Si bien el acceso abierto al conocimiento científico constituye un principio ampliamente respaldado por la comunidad académica y los organismos internacionales, su implementación ha estado dominada por el cobro para el procesamiento de artículos (*Article Processing Charges - APC*). Este modelo ha trasladado el costo de la publicación a los autores o sus instituciones, profundizando las desigualdades entre países e instituciones con diferentes niveles de recursos ^4^^,^^5^.

La concentración del mercado editorial en un número reducido de corporaciones multinacionales ha reforzado estas presiones. En este escenario, el acceso, la visibilidad y el prestigio científico se encuentran mediados por lógicas comerciales que no siempre se alinean con las prioridades de la salud pública ni con las necesidades de los países de ingresos medios y bajos.

Un desafío emergente que pone a prueba aún más el sistema editorial es el uso creciente de herramientas de inteligencia artificial (IA) en la producción científica. La IA generativa puede apoyar tareas de redacción o traducción, pero su uso no regulado ha facilitado la generación de manuscritos con errores conceptuales, referencias inexistentes y opacidad en la autoría. Asimismo, el uso de IA en la revisión por pares plantea interrogantes sobre sesgos algorítmicos y responsabilidad editorial ^6^.

Desde la perspectiva de la salud pública, el riesgo no radica en la tecnología en sí, sino en su uso acrítico dentro de un sistema ya tensionado por incentivos inadecuados. Frente a ello, resulta indispensable avanzar hacia un uso razonable y transparente de la IA ^(^^6^.

De otro lado en el contexto peruano, se ofrece múltiples ejemplos de la falta de equivalencia entre el impacto sanitario real y la visibilidad bibliométrica. Los estudios sobre hepatitis B y D desarrollados en la Amazonía y zonas altoandinas contribuyeron de manera decisiva a políticas de vacunación universal y control de la transmisión horizontal y vertical, alineadas con las recomendaciones de la OMS ^7^^,^^8^.

Durante la pandemia de COVID-19, el desarrollo y validación de pruebas diagnósticas permitió ampliar la capacidad diagnóstica y fortalecer la vigilancia epidemiológica en tiempo real ^9^. Avances en diagnóstico de arbovirosis como dengue, anemia y exposición a metales pesados han sustentado acciones de salud pública orientadas a la equidad territorial y ambiental ^10^^-^^12^.

De manera similar, la investigación sobre resistencia a antimaláricos orientó cambios terapéuticos y fortaleció la vigilancia de la eficacia farmacológica frente a la malaria en la Amazonía peruana ^13^. Los estudios sobre tuberculosis contribuyeron al ajuste normativo y a la respuesta programática nacional ^(^^14^ al igual que en VIH/SIDA ^15^.

Estos ejemplos muestran que el impacto de la investigación en salud pública adopta formas normativas, programáticas y diagnósticas que no siempre son capturadas por métricas bibliométricas tradicionales.

Frente a este escenario, se propone una evaluación dual de las revistas científicas, que complemente indicadores bibliométricos con métricas de impacto en salud pública, en línea con iniciativas internacionales de reforma de la evaluación de la investigación ^16^.

La visibilidad internacional sigue siendo un medio legítimo y necesario para amplificar la circulación del conocimiento científico. Sin embargo, el fin último de la ciencia en salud pública debe ser mejorar la salud de las poblaciones y reducir inequidades, integrando excelencia editorial, integridad científica y uso responsable de nuevas tecnologías.
